# Central Dental Association of New Jersey

**Published:** 1891-10

**Authors:** 


### Central Dental Association of Northern New Jersey.

NEWARK, N. J., May 8th, 1891.

At a meeting of the Central Dental Association of Northern New Jersey, held April 20th, 1891, the following resolutions were unanimously adopted, and a motion was carried that a copy be sent to the bereaved family, and sent to each of the Dental journals to be published. Respectfully,

S. S. HAWLEY, Sec'y.

We, the members of the Central Dental Association of Northern New Jersey, having learned with sincere regret of the death of our friend and fellow-member, W. H. Atkinson, M.D., D.D. S., of New York, who by reason of his great abilities, scholarship, zeal, industry and self-sacrificing devotion to the interests of the dental profession, and the never-failing willingness to impart his knowledge to all who ask it, he was recognized by us as the most influential member of our profession, a man who devoted his life to its honor and advancement.

During the eleven years of the existence of this Society, he has scarcely missed a meeting, and his relations with us have been such, that it is our pleasure and duty to record our high appreciation of him.

That by the death of Dr. Atkinson the dental profession has been deprived of one of its most able and useful members, one whose influence for good will last while dentistry exists.

We have lost one of our best friends, and as we fondly called him "Father Atkinson," so indeed do we feel we have lost our "Father in Dentistry."

We, therefore, extend to his family, and to our brother members, of the dental profession, our sincere sympathy in their great bereavement, and that a copy of these resolutions be sent to the family of the deceased, and that they also be published in the Dental journals.

[SIGNED.]

J. ALLEN OSMUN,<br>

C. S. STOCKTON<br>

CHAS. A. MEEKER,<br>

S. C. G. WATKINS,<br>

C. W. HOLBROOK.<br>

} COMMITTEE.

